# Changes in Plasma Free Fatty Acids Associated with Type-2 Diabetes

**DOI:** 10.3390/nu11092022

**Published:** 2019-08-28

**Authors:** Amélie I. S. Sobczak, Claudia A. Blindauer, Alan J. Stewart

**Affiliations:** 1 School of Medicine, University of St Andrews, St Andrews KY16 9TF, UK; 2 Department of Chemistry, University of Warwick, Coventry CV4 7EQ, UK

**Keywords:** cardiovascular disease, fibrates, free fatty acids, lipidomics, metabolism, statins, type-2 diabetes mellitus

## Abstract

Type 2 diabetes mellitus (T2DM) is associated with increased total plasma free fatty acid (FFA) concentrations and an elevated risk of cardiovascular disease. The exact mechanisms by which the plasma FFA profile of subjects with T2DM changes is unclear, but it is thought that dietary fats and changes to lipid metabolism are likely to contribute. Therefore, establishing the changes in concentrations of specific FFAs in an individual’s plasma is important. Each type of FFA has different effects on physiological processes, including the regulation of lipolysis and lipogenesis in adipose tissue, inflammation, endocrine signalling and the composition and properties of cellular membranes. Alterations in such processes due to altered plasma FFA concentrations/profiles can potentially result in the development of insulin resistance and coagulatory defects. Finally, fibrates and statins, lipid-regulating drugs prescribed to subjects with T2DM, are also thought to exert part of their beneficial effects by impacting on plasma FFA concentrations. Thus, it is also interesting to consider their effects on the concentration of FFAs in plasma. Collectively, we review how FFAs are altered in T2DM and explore the likely downstream physiological and pathological implications of such changes.

## 1. Introduction

In 2017, the worldwide occurrence of diabetes (both type-1 and type-2 diabetes mellitus (T2DM)) was estimated to be at 425 million individuals, with this number predicted to rise to 629 million by 2045 [1]. Diabetes is characterised by a mishandling of glucose levels in the blood through defective insulin signalling. In T2DM, cells in the body become resistant to insulin (often due to defects in insulin receptor functioning) and fail to properly respond. Thus, blood insulin levels are mostly either unchanged or elevated in individuals with T2DM. However, T2DM can also evolve into insulin deficiency through a loss of the insulin storage and secretion function of the β-cells of the pancreas. Altered blood glucose levels have important consequences in the body. Indeed, they are directly or indirectly associated with many physiological processes including the control of glycogen and lipid metabolism, the control of food intake (satiety), maintenance of body weight and the regulation of inflammation, vasodilatation and basic cell growth and replication. Thus, a dysregulation of glucose levels has wide-ranging consequences in the body. As the metabolism of fats and carbohydrates are intimately intertwined, altered free fatty acid (FFA) levels in plasma and their metabolism are both a cause [2,3] and a consequence [4] of insulin resistance and T2DM, with many deleterious downstream effects such as chronic inflammation, loss of pancreatic β-cells, atherosclerosis, and heart disease being caused or exacerbated by elevated FFAs.

In healthy individuals, FFA metabolism is tightly regulated. This is necessary because fatty acids do not only serve as efficient stores of energy in the form of triacylglycerides (TAGs) in adipocytes and constituents of all cellular membranes, primarily in the form of phospholipids. Crucially, FFAs are also the primary fuel for heart and skeletal muscles and are precursors of hormones and non-hormonal signalling molecules. Several disorders (including T2DM) are associated with obesity, which leads to excess fat storage and dysregulated adipocyte signalling, causing a variety of pathologies [5]. This review will examine the links between T2DM and the FFAs present in plasma of individuals with T2DM and how this affects FFA metabolism. The effect of lipid-lowering drugs on plasma FFA levels and FFA-induced complications will also be surveyed. For Section 3, Section 4 and Section 5, we have searched the PubMed database using appropriate combinations of “FFA OR free fatty acid OR NEFA OR non esterified fatty acid”, “T2DM OR type 2 diabetes OR type II diabetes OR type-2 diabetes OR type-II diabetes”, “diet or dietary” (Section 3 only), “plasma OR blood”, and “levels OR concentrations” (for Table 1 and Table 2). For Section 6, “T2DM and FFA” (including alternative expressions as shown above) were combined with “lipid lowering drugs” as well as the names of the individual drugs and agents listed in Table 3 and Table 4. For Section 2, further authoritative reviews and primary sources were consulted and cited, to provide the appropriate background information.

## 2. FFA Metabolism in Healthy Tissues

### 2.1. Origins of Plasma FFAs

Fatty acids (FAs) are mainly ingested in the form of phospholipids and TAGs. During digestion, TAGs are hydrolysed into mono- and diglycerides and free fatty acids (FFAs) [6]. Only short- and medium-chain FFAs (up to 12 carbons) can be absorbed by enterocytes directly and, after transfer into the bloodstream, are transported by serum albumin, the most important extracellular FFA transporter [6]. Long-chain FFAs (over 12 carbons) are reconverted to TAGs after absorption and are transported in lipoprotein particles [6]. Lipoprotein particles are classified into different groups depending on their size, including HDL, LDL, very low-density lipoprotein (VLDL) and chylomicrons (also called ultra-low-density lipoproteins). The latter are the main plasma vehicle for dietary TAGs [6]. Lipoprotein lipase present at the surface of cells cleaves TAGs to form FFAs that are taken up by cells [7]. Uptake is mediated by various transmembrane proteins (CD36, FATP2, FATP4, and FATP5). Cleavage and uptake occurs in most cells of the body, but is particularly important in adipose tissue, where FFAs are re-esterified to form TAGs that are stored in the fat droplets of adipocytes [8]. However, when TAGs in the chylomicrons are cleaved, the resulting FFAs are not always directly taken up by the nearby cells [9]. In such cases, these “spill-over” FFAs instead bind to serum albumin and are transported through the circulation to other cells [9].

Another source of plasma FFAs is their endogenous synthesis from (excess) carbohydrates, in a process termed de novo lipogenesis [7,8]. This can occur in most cells, but is particularly important in the liver, adipose tissue and the mammary glands. De novo lipogenesis is upregulated by insulin in the blood and downregulated by high levels of the hormones adrenaline and glucagon [7]. FFA synthesis utilises acetyl-CoA units derived from citrate, eventually giving palmitic acid (C16:0), which can be further elongated (e.g., to stearic acid; C18:0) or desaturated (e.g., to palmitoleic acid; C16:1n-7) to form other FFAs [7] (although FFAs are expected to be deprotonated and hence anionic at neutral pH, we retain common nomenclature where only esterified fatty acids are referred to with the suffix “-ate”). Those FFAs are then converted into TAGs [7]. The liver also synthesises VLDL, which then transports the newly synthesised TAGs via the blood to other tissues including adipose tissue [7]. Fatty acids stored as TAG in adipocytes can be mobilised by lipolysis, i.e., the hydrolysis of the TAGs [8]. In the bloodstream, the now free FAs are again primarily carried by serum albumin [8]. Lipolysis is enhanced when plasma levels of insulin are low and levels of catecholamines are high [5,8,10,11], for example after fasting or during prolonged exercise. This ensures a steady basal supply of fuel for skeletal muscles and the heart muscle independent of food intake [12].

Once taken up by cells or synthesised by de novo lipogenesis, most FFAs are transported by intracellular fatty-acid binding proteins (FABPs) to the mitochondria where they undergo β-oxidation to release energy in the form of ATP [8,10]. However, FFAs can also be converted to phospholipids and sphingolipids to participate in forming cellular membranes. FFAs are also important signalling molecules [13]. For example, certain fatty acids within membrane phospholipids can be cleaved by phospholipase A2 isoforms and converted to form inflammatory mediators (called eicosanoids or prostanoids) in the immediate vicinity of the cells [14].

### 2.2. Regulation of FFA Metabolism: FFA Receptors

To maintain homeostasis, FFAs, like other nutrients, must be sensed by specific proteins [15], which triggers a range of downstream effects. FFAs bind to several cell-surface receptors of the GPCR family, including GPR40, GPR43, GPR41, GPR120, and GPR84 [16,17]. Long-chain FFAs are ligands for GPR40 (also called free fatty acid receptor 1, FFA1) [18] and GPR120 (also called FFA4) [19,20], whilst GPR41 and 43 (FFA2 and FFA3) are short-chain FFA sensors. Ligand binding typically triggers an increase in cytosolic calcium, with further downstream effects that differ between the FFA receptors. Their expression profiles also differ, but they are expressed in a variety of tissues and cells, including enteroendocrine cells, the brain, adipocytes, pancreatic β-cells, and immune cells. In the gut, GPR40 and GPR120 are proposed to sense dietary fat, affecting energy homeostasis indirectly via hormonal signalling. Amongst other pathways, this involves stimulating the secretion of incretins [16] such as glucagon-like peptide-1, which in turn increases insulin secretion by β-cells [21]. They may also mediate effects on appetite and uptake of energy from food [22]. In pancreatic β-cells, long-chain FFA binding to GPR40 increases glucose-stimulated insulin secretion [18], presumably to promote uptake of energy-rich molecules (carbohydrate- and fat-derived) after a meal by liver and adipose tissue. These findings may not only furnish one of the many links between diet and T2DM prevalence (see Section 3), but also explain why GPR40 and GPR120 are under intense study as drug targets. The short-chain FFA receptors GPR41 and GPR43 are of interest in the context of gut microbiome-mediated effects on metabolic health [13], as gut bacteria provide a major source of short-chain FAs (SCFAs; see Section 5.2.2). The latter insights offer novel approaches to influence these sensors, and the physiological effects they mediate, through diet [16]. 

It has been proposed that different types of FFA may exert differential effects on some of these receptors. Specifically, long-chain n-3 FFAs (such as eicosapentaenoic acid (EPA; C20: 5n-3) and docosa-hexaenoic acid (DHA; C22:6n-3)) have been shown to activate GPR120 in enteroendocrine L-cells, adipocytes and pro-inflammatory macrophages to reduce inflammation [23]. In this case, the signalling pathway occurs through the activation of β-arrestin-2 which prevents growth factor β-activated kinase 1 (TAK1) from binding to the TAK1-binding protein, TAB1 [23]. Notably, the ability of long-chain FFAs to bind to GPR40 or GPR120 is affected by the presence of serum albumin [16], as receptors and albumin compete for the same ligands. It can be anticipated that this competition depends on the type and length of FFA.

Another important membrane-bound protein for FFA sensing is the CD36 fatty acid translocase (FAT) protein [24]. CD36 is a multifunctional scavenger receptor, amongst other entities for oxidized phospholipids [25]. It is expressed in many cell types including enterocytes, myocytes, macrophages, endothelial cells, and platelets, and is of particular importance in the context of the effects of dyslipidaemia on inflammation and cardiovascular health [26] (see Section 5). 

Once inside a cell, long-chain FFA catabolism starts in peroxisomes [7], and this is also associated with FFA signalling. Peroxisome proliferator-activated receptors (PPAR) are ligand-activated nuclear transcription factors that bind and respond to long-chain FFAs, as well as to eicosanoids [17,27]. Their binding pockets are relatively large and thus accommodate a variety of FFAs beside other molecules [28].

PPARα binds both saturated and unsaturated FFAs, regulates FFA uptake and oxidation and inhibits de novo fatty acid synthesis; it is predominantly expressed in tissues that possess a high fatty acid catabolism (e.g., liver, kidney, heart and skeletal muscles) [15,17,28]. PPARγ responds in particular to unsaturated FFAs and various eicosanoids, prostaglandins and related molecules [28]. It regulates the uptake and storage of FFAs and glucose homeostasis mostly in adipose tissue, but also in skeletal muscle. PPARγ is one of the critical links between FFA metabolism, signalling and inflammation: many of its ligands are involved in the regulation of inflammation; their binding activates PPARγ, which ultimately reduces inflammation [28]. PPARγ is also expressed in macrophages and induces these cells to differentiate into the non-inflammatory M2 type [29]. PPARδ is the most abundant form of these receptors in skeletal muscle and mediates metabolic changes in response to fasting and prolonged exercise [17]. All of these FFA receptors are the targets of existing and novel drugs for the prevention or treatment of T2DM, its risk factors and/or its downstream complications (see Section 6).

## 3. Associations between the Intake of Fatty Acids and Occurrence of T2DM

The causes of T2DM are complex; however, among the range of different factors, the contribution of diet is undisputed [30], with intake of too many calories and the resulting obesity being clear risk factors [31,32]. However, the case for or against fatty acids is less clear-cut than one may expect. Numerous recent studies, systematic reviews and meta-analyses have investigated the association between total amounts, type, and origins of dietary fats and T2DM [32,33,34,35,36,37]. Most of these studies are observational, with a scarcity of interventional studies such as randomly-controlled clinical trials (RCTs). There are inherent difficulties in trying to establish the effects of an individual nutrient (such as an individual fatty acid) contained in a normal diet. Most observational studies use food diaries and/or retrospective reports, and the associated “human error” has been highlighted frequently. Alternatively, plasma (and tissue) FAs can be directly determined quantitatively by analytical methods. We note that many such studies do not focus on FFAs, but on total FAs which include those present in TAGs and phospholipids. Furthermore, it is critically important to consider sources of circulating plasma (F)FAs [6,38] (see Section 2), as they may not always reflect dietary intake. Especially after a meal, the FAs ingested may constitute up to 50% of circulating FFAs [39], as some of the de-esterified FFAs are released into plasma (“spill-over”) [38]. At other times, lipolysis and de novo lipogenesis play major roles in defining the composition of circulating FFAs.

Taking these methodological limitations and constraints into account, newly diagnosed and undiagnosed diabetics have been found to have higher intakes of total and saturated fat than healthy controls [33,40]. However, recent large-cohort studies and comprehensive reviews of observational and interventional studies have found no association between the total fat dietary intake (as a percentage of total calories) and incidence of T2DM [34,37,41,42]. There is, in fact, no compelling evidence to support the notion that low-fat diets are beneficial for the avoidance of metabolic syndrome and T2DM [34,37,43], and there have been calls for public health recommendations and nutritional policies to be updated accordingly [34].

Several recent reviews consider associations between T2DM risk and the type of fat [32,34,44]. The latter has been suspected to be of significance as far back as 1959 [45]. Primarily, apart from cholesterol, the type of fat is defined by its FAs: the major division concerns saturated (SFAs) and unsaturated FAs, and the latter can be further divided into mono- (MUFAs) and poly-unsaturated acids (PUFAs). The types of fatty acids present in various food groups are thought to play a pivotal role in whether or not such food is considered beneficial, neutral, or detrimental with respect to developing or living with T2DM [33], but information available from numerous cohort studies is often conflicting.

Although there is a persistent notion that SFAs should generally be avoided, the scientific case for this recommendation in conjunction to the risk of developing T2DM is not entirely clear-cut [37,43,46]. To some degree, this is due to the fact that not every SFA has the same biological effects, but also to the fact that humans do not consume specific nutrients in isolation, but as part of more or less complex foods that contain other nutrients that may exert significant influence on whether consumption of a particular source of FA is detrimental or beneficial [34,47]. Dairy products, rich in SFAs, may be a case in point [36,48,49,50,51,52,53]. Consumption of yoghurt has overwhelmingly been found to be associated with a lower risk of T2DM, irrespective of fat content. The case for cheese is less clear, most likely due to the diversity of what is classified as “cheese”, but it appears that fermented cheese is likely beneficial [7], whilst unfermented cream cheese has been associated with increased T2DM risk [54]. Milk consumption is not clearly associated with T2DM risk, whereas butter consumption [55] and a high intake of full-fat dairy-based products [47] have been seen to associate with lower T2DM incidence. Whilst these and related studies [32,34] suggest on the one hand that there is no scientific case for avoiding full-fat dairy [34,36,51], it has also been proposed that dairy fat as such has no beneficial effects [33]. The fairly clearly observed inverse correlations of dairy consumption have been ascribed to the simultaneous consumption of the fat-soluble vitamins D and K2, both of which are associated with decreased T2DM incidence [56,57,58], as well as to interactions of the probiotics contained in fermented dairy with the gut microbiome [34]. It may be noted that these suggestions still hold for studies where inverse correlations between plasma biomarkers for dairy consumption such as the SFAs pentadecanoic and heptadecanoic acid (C15:0 and C17:0, respectively) (and their phosphoryl esters) and T2DM risk were found [59,60]. It may also be cautioned that the validity of these SFAs as biomarkers for dairy fat intake has been questioned [61], as they also occur in red meat and many other animal- or plant-based foods, or may be endogenously produced. What is clear (from food diaries/questionnaire-based studies) is that SFAs derived from meat associate with increased T2DM risk, although it remains to be seen whether this is not exclusively due to other components [34].

A further aspect to consider for SFAs is chain length. Although circulating plasma and tissue long even-chain SFA (C14:0, C16:0 and C18:0) levels are clearly associated with T2DM risk [33,34], intake of C16:0 and C18:0 (abundant in meat, coconut oil, and hard cheeses) is not necessarily, and intake of C14:0 was in fact associated with reduced risk in at least one study [47]. It follows that increased plasma (and tissue) even-chain SFAs in obesity and T2DM are most likely predominantly a result of lipolysis and, perhaps even more importantly, lipogenesis from excess carbohydrates, which principally yields even-chain SFAs. In contrast, intake of shorter SFAs (e.g., myristate, laurate and even shorter ones (chain lengths of C4-10)), as well as odd-chain SFAs (C15:0 and C17:0 and their phosphoryl esters), all predominantly derived from dairy products, correlated with lower T2DM risk [47]. Apart from the latter study, relatively little attention has been dedicated to dietary medium-chain FAs (MCFAs) so far, but a recent pilot clinical trial [62] indicated that high MCFA intake is worthy of further exploration for combating cardiomyopathy in T2DM. Similarly, circulating levels of very long-chain SFAs (arachidic acid (20:0), behenic acid (22:0), and lignoceric acid (24:0)), which occur in various nuts but can also be produced endogenously through chain elongation, were each associated with reduced T2DM risk [63]. Whether these associations are again a consequence of other components present in the respective food sources or reflect direct effects of these fatty acids is unknown.

The degree of desaturation has also been considered in several recent studies and reviews. The most abundant MUFA in typical diets is oleic acid (OA; C18:1n-9). Glycerol esters of OA are the major components of many vegetable oils, most prominently olive oil. Evidence regarding effects of MUFAs on T2DM risk is somewhat mixed. A seminal clinical trial in 2001 suggested that replacing MUFAs with SFAs had adverse outcomes [64], and a recent meta-analysis of RCTs suggested that diets high in MUFAs resulted in lower fasting glucose than diets high in carbohydrates or PUFAs [65]. Similar beneficial effects have been observed in several other studies [32,66], but other recent reviews report no associations with diabetes incidence [34,67]. It has been suggested that the consumption of certain foods rich in OA (such as extra-virgin olive oil, rapeseed oil) may be beneficial due to other components such as polyphenols.

Important dietary PUFAs include the essential linoleic acid (LA; C18:2n-6) and α-linolenic acid (ALA; C18:3n-3), and further n-3 FAs predominantly derived from marine sources such as eicosapentaenoic acid (EPA; C20:5n-3) and docosahexaenoic acid (DHA; C22:6n-3). PUFAs in general, and n-3 PUFAs in particular, enjoy a widespread reputation of being healthy fats, and several studies have also highlighted positive effects with regard to T2DM risk or biomarkers [44,67,68], especially for n-3 PUFAs [69]. However, a very recent “umbrella” review of intervention studies with n-3 PUFAs that focused on glycaemic control found the overall available evidence to be inconclusive [70], irrespective of whether the PUFAs were of plant or marine origin. There also appears to be a dependence on sex and genetic background. Systematic reviews found that treatment with n-3 PUFAs (including ALA, EPA, DHA, administered in a variety of ways) benefited women only [71], whilst Asians benefited in both interventional [68] and observational [72] studies. In contrast, marine long-chain n-3 FAs consumption in fact appeared to increase T2DM risk in Americans in general [72] or American women [73]—even though in a related study high plasma levels of the same FFAs were associated with decreased risk [16]. Studies and systematic reviews are also available for specific dietary PUFAs. The Singapore Chinese Health Study (43,176 Chinese men and women) inspected marine and non-marine n-3 and n-6 intake and recorded that a decreased T2DM risk was associated with ALA consumption [74]. Similarly, the European Prospective Investigation into Cancer and Nutrition (EPIC)-InterAct Consortium found in a large case-cohort study (12,132 cases and 15,919 sub-cohort participants) that ALA, measured as plasma phospholipid, was inversely associated with T2DM [75]. Conversely, a systematic review of RCTs on ALA supplementation did not reveal any correlations with T2DM markers [76]. In the EPIC-InterAct study, the n-3 PUFAs EPA and DHA were not associated with T2DM [75].

There have been suggestions that the n-6:n-3 ratio plays a role in T2DM development [77,78]; these are mainly based on animal studies exploring the hypothesis that n-6 PUFAs may be pro-inflammatory, and hence may promote insulin resistance (see Section 5). However, for humans, there is no convincing epidemiological or clinical evidence that would support recommendations to lower dietary intake of n-6 PUFAs [33,75]. On the contrary, higher intake or increased plasma levels of n-6 LA have been shown to be associated with lower T2DM risk [33,67,75]. Eicosadienoic acid (C20:2n-6) was also inversely associated with T2DM incidence, whilst no association was found for arachidonic acid (ARA; C20:4n-6). In contrast, plasma levels of γ-linolenic acid (GLA), dihomo-GLA, docosatetraenoic acid (DTA), and docosapentaenoic acid (DPA; C22:5n-6) were associated with higher T2DM incidence [75]. The latter are all metabolites of LA [79].

All MUFAs and PUFAs considered so far contain exclusively *cis*-double bonds. There are natural *trans*-FAs, e.g., *trans*-palmitoleate (C16:1 *trans*-n-7), present in ruminant milk and dairy products. Like the odd-chain SFAs C15:0 and C17:0, *trans*-palmitoleate has been used as a plasma biomarker for dairy consumption and has been negatively associated with incidence of T2DM in several studies [80,81,82]. However, most *trans*-FAs in the diet have been products of (industrial) processes such as frying and baking, and—before the introduction of improved regulations and processes—partial hydrogenation of vegetable oils, giving margarine or “spreads” meant to replace butter. The evidence for harmful effects of non-natural *trans*-FAs has been quite clear [83], and elevated *trans*-FA levels in the diet have also been associated with T2DM [84]. This may be due to the fact that they adversely affect plasma lipid profiles (raising total cholesterol, LDL cholesterol and TAG levels, and reducing HDL cholesterol levels) as well as causing systemic inflammation, and dysregulated endothelial function [85]. More recent studies have been unable to find any association between *trans-*FA intake and incidence of T2DM [35,43,86]; this is likely due to the now much reduced levels of industrially produced *trans*-FAs in the Western diet.

Finally, it is worth noting that the “Mediterranean Diet”, characterised by relatively high fat intake in the form of olive oil, nuts, and seafood, seems to decrease the incidence of T2DM [49,87]. It is however not yet possible to say whether the types of fat consumed in this diet (high percentage of MUFAs and PUFAs) is a contributing factor to this association, or whether other food components are more significant [87].

## 4. Differences in Plasma FFAs in T2DM

Although many of the specific associations between the intake of particular FFAs and incidence of T2DM require further study, the changes in plasma FFA levels observed in subjects with T2DM are clearer. Numerous studies have highlighted that T2DM patients tend to have elevated total plasma FFAs [88,89,90], and that impaired insulin secretion, impaired insulin sensitivity and glucose intolerance are all strongly associated with elevated plasma FFA levels, especially for saturated FFAs (including palmitic and stearic acid) [33,91]. In addition, levels of palmitic acid are positively correlated with levels of glycated haemoglobin (HbA1c) in T2DM subjects, while levels of the unsaturated FFA OA were only correlated to HbA1c levels in patients with inadequate diabetes control [92].

As indicated previously, plasma FFA concentrations not only reflect FFA intake, but also the balance between de novo FFA synthesis, storage as TAGs and lipolysis of these TAGs. Therefore determination of FFA intake is insufficient to understand the link between plasma FFAs and T2DM. Lipidomic analyses have been performed on blood samples taken from T2DM cohorts; however such studies have mostly focused on the quantification of different lipid types (glycerol esters or phospholipids) or on the FA composition of different cell membranes rather than on FFAs [93]. For example, the FA profiles of erythrocyte and leukocyte membranes have been determined, showing that individuals with T2DM have a higher palmitate content in isolated erythrocyte membranes (25.4 ± 3.1% in controls and 31.1 ± 2.4% in individuals with T2DM, *p* < 0.005) but not in leukocyte membranes (29.3 ± 2.4% in controls and 29.3 ± 5.2% in individuals with T2DM, *p* < 0.01) [94]. Individuals with T2DM also had a higher SFA/unsaturated FA ratio in those membranes compared to controls (for erythrocyte membrane, 0.72 ± 0.07 in controls vs 0.97 ± 0.06 in individuals with T2DM, *p* < 0.001; for leukocyte membrane, 0.83 ± 0.12 in controls vs 1.12 ± 0.14 in individuals with T2DM, *p* < 0.001). These changes in the fatty acid composition of cell membranes have a direct impact on the fluidity of those cells [95]. Some studies also focus on phospholipid FAs in plasma. In one such study, high plasma levels of long-chain n-3 FAs and ALA were associated with lower T2DM risk [96], but it is worth noting that the same study (Women’s Health Study) found that consumption of long-chain (marine) n-3 PUFAs in fact increased the risk of T2DM [73]—highlighting once more that the correlation between plasma FAs and FAs in the diet is not straightforward.

Nevertheless, a number of studies have directly examined T2DM-related changes in the plasma concentrations of individual FFAs. Those studies and the characteristics of the populations investigated are summarised in Table 1, while the corresponding results are presented in Table 2. 

**Table 1:** Studies having measured the concentrations of individual FFA species in the blood from subjects with T2DM and controls and characteristics of the populations studied.

References	Numbers of Subjects		Population	Type of Results
	Ctrl.	T2DM		
Clore et al., 2002 [97]	6	6	BMI- and age-matched; not sex-matched; American cohort	Plasma FFA conc. after a 14 h fast.
Yi et al., 2007 [98]	45	78	Age-, sex- or BMI-matched; Chinese cohort	Plasma FFA conc., fasting not indicated.
Liu et al., 2010 [99]	50	53	Age-and sex-matched; not BMI-matched; Chinese cohort	Plasma FFA conc. after fasting.
Grapov et al., 2012 [100]	12	43	BMI-matched; not age-matched; obese African-American women	Geometric mean of plasma FFA after an overnight fast
Lu et al., 2016 [101]	197	197	Age-and sex-matched; not BMI-matched; Chinese cohort	Trend of plasma FFA, no fasting.
Ma et al., 2018-a [102]	40	21	Age-and BMI-matched; not sex-matched; Kazakh cohort	% total plasma FFA after an 8 h fast.
Ma et al., 2018-b [102]	35	39	Not age-, BMI- or sex-matched; Uyghur cohort	% total plasma FFA after an 8 h fast.

**Table 2:** Changes in FFA concentrations measured in blood from subjects with T2DM compared to controls. Elevated FFA concentrations are indicated with ↑, reduced concentrations with ↓, unchanged concentrations with =.

FFA Species	Alterations in Plasma FFA Conc. in T2DM						
	Clore et al., 2002 [97]	Yi et al., 2007 [98]	Liu et al., 2010 [99]	Grapov et al., 2012 [100]	Lu et al., 2016 [101]	Ma et al., 2018-a [102]	Ma et al., 2018-b [102]
Total FFA			↑	↑			
Saturated FFA	↑			↑			
C6:0, caproic acid						=	↑
C8:0, caprylic acid						=	=
C10:0, capric acid						=	=
C12:0, lauric acid		=				=	=
C14:0, myristic acid	=	↑	↑	↑		=	=
C15:0, pentadecanoic acid		↑				=	=
C16:0, palmitic acid	↑	↑	↑	↑	↑	=	=
C18:0, stearic acid	↑	↑	↑	↑	↑	=	=
C19:0, nonadecylic acid				↑			
C20:0, arachidic acid		↑		↑		=	=
C22:0, behenic acid						=	↓
C24:0, lignoceric acid		=	↑	=			
Unsaturated FFA				↑			
Monounsaturated FFA				↑			
C14:1n-9, myristoleic acid						↓	↓
C16:1n-7, palmitoleic acid	=	↑	↑	↑↑		=	=
C16:1n-9, *cis*-7 hexadecenoic acid		↑					
C18:1n-9, oleic acid (OA)	↑	↑	↑	↑	↑	=	=
C18:1 *trans*-n-7, vaccenic acid		↑		↑			
C19:1n-9, *cis*-10 nonadecenoic acid				=			
C20:1n-9, gondoic acid				↑			
C24:1n-9, nervonic acid			↑				
Total n-7				↑			
Total n-9				↑			
Polyunsaturated FFA	=			↑			
C18:2n-6, linoleic acid (LA)	=	↑	↑	↑	↑	=	=
C18:2 *trans*-n-7 *cis*-n-9, rumenic acid				↑			
C18:3n-6, γ-linolenic acid (GLA)		↑	↑	=		↓	↓
C18:3n-3, α-linolenic acid (ALA)	=	=	↑	↑		=	=
C20:2n-6, eicosadienoic acid			=	=		=	=
C20:3n-6, dihomo-γ-linolenic acid (DGLA)		↑		=		↓	↓
C20:4n-6, arachidonic acid (ARA)	=	↑	↑	=		=	=
C20:5n-3, eicosapentaenoic acid (EPA)	=	↑	↑	=		=	↑
C22:4n-6, adrenic acid		↑		↑			
C22:5n-3, docosapentaenoic acid		=	↑	↑			
C22:5n-6, osbondic acid	=			=			
C22:6n-3, docosahexaenoic acid (DHA)	=	↑	↑	=		=	=
*Trans* FFA				↑			
C16:1 *trans*-n-7*, trans*-palmitoleic acid				↑			
C18:2 *trans-*n-6, linolelaidic acid				↑			

The data are heterogeneous, which can be explained by the different ethnicities of the populations studied, by the difference in the controls selected, in particular if they were matched in BMI or not, by whether fasting was required before blood collection or not, and by whether the results are presented as absolute concentrations or the percentage of the total plasma FFA measured. With the exception of the Ma et al. study [102] (carried out on Kazakh and Uyghur populations), the plasma FFA concentrations measured in the T2DM cohorts were all either unchanged or increased compared to the controls, whilst some FFAs were decreased in the Ma et al. study. This might be attributable to the ethnicity or diet of the populations included. Globally, the two studies that reported total FFA concentrations (Liu et al. and Grapov et al.) found this parameter to be increased in the T2DM cohort, a frequent observation made previously [88,89,90]). Similarly, the two studies that examined saturated FFA concentrations (Grapov et al. and Clore et al.) detected an increase of these in the T2DM cohort. Grapov et al. also reported total unsaturated FFA, mono-unsaturated FFA, polyunsaturated FFA and *trans*-FFA concentrations, and found them all to be increased in the T2DM cohort. This is particularly meaningful, as this study was carried out on controls and a T2DM cohort matched for BMI. The Clore et al. study also reported polyunsaturated FFA concentrations but did not detect any change between the T2DM cohort and the controls.

There are some common trends for individual FFAs in five out of the six studies (Liu et al. [99], Grapov et al. [100], Lu et al. [101], Yi et al. [98] and Clore et al. [97]), namely an increase in the SFAs palmitic and stearic acid, and the MUFA OA. This common feature is even evident in the studies without BMI-matched controls (i.e., Clore et al., Yi et al., and Lu et al.). LA was also increased in four out of the six studies where it was measured, and palmitoleic acid was raised in three studies. The heterogeneity observed for the remainder of the FFAs may be a consequence of small cohort size, appropriateness of controls, variation in diets and/or the genetic backgrounds of the populations studied, as well as the sensitivity of the analytical method used.

Apart from these small cohort case-control studies, some data are also available from cross-sectional or prospective studies. In older Finnish men, total FFA, SFAs, MUFAs, n-7 and n-9 FFAs in fasting serum correlated with increased blood glucose and T2DM risk, whilst high levels of n-6 FFAs, primarily LA, correlated with lower T2DM risk [90]. Total n-6 FFAs, LA and ARA were also associated with lower T2DM risk in a prospective Finnish study of 2189 men [103]. Similarly, glucose tolerance was better in women after gestational diabetes where they had higher levels of LA [104]. In a study with 667 participants, LA associated with low fasting glucose in cross-sectional but not prospective analyses [105]. A recent review has concluded that “biomarkers of LA intake are associated with reduced risk of T2DM and better glycaemic control and/or insulin sensitivity” [67]. 

Summarily, the most common pattern collectively observed in individuals with T2DM in case-control, cross-sectional and prospective studies was an elevation in saturated FFAs (in particular palmitic acid). Evidence regarding other FFAs is often inconclusive, illustrated by associations for LA, where large cross-sectional studies highlight an inverse relationship with T2DM risk, whilst the small case-control studies summarised in Table 2 indicated that LA levels were higher in T2DM patients than in controls. Thus, changes in the concentrations of individual FFAs (or types of FFAs) associated with T2DM are complex and under-studied. The need for further investigation is also illustrated by a recent non-targeted case-control metabolomic study, which found that out of 42 potential biomarkers for T2DM, 12 were plasma FFAs [106]. Two long-chain n-6 FFAs (adrenic acid (C22:4n-6) and ARA) were elevated in patients with impaired fasting glucose, whilst some medium/short-chain FFAs (pelargonic acid (C9:0), heptanoic acid (C7:0) and the MUFA 5-dodecenic acid (C12:1n-7)) were decreased in these patients.

Next, the potential effects of these alterations in plasma FFA concentrations will be examined.

## 5. FFA Metabolism in T2DM

The frequently observed elevated levels of plasma FFAs in obese (and T2DM) patients result in metabolic changes, which can lead to numerous pathologies including insulin resistance and T2DM itself [4]. Fat is normally stored in adipocytes in the form of TAGs, but when plasma FFAs levels are too high, the adipocytes become overwhelmed. As a consequence, fat accumulates in the cells of other organs as lipid droplets, and this can cause lipotoxicity under certain circumstances [10,107]. In addition, FFAs are directly and indirectly linked to the production of inflammatory molecules and the regulation of inflammation [108,109], a common co-morbidity of T2DM. Dyslipidaemia may also affect cellular membranes, which may impair cellular functions [110]. All these aspects have been proposed as contributing factors to FFA-induced insulin resistance, and will be further discussed in the following sections, also paying attention to differential effects of different types of FFAs.

**Figure 1:**
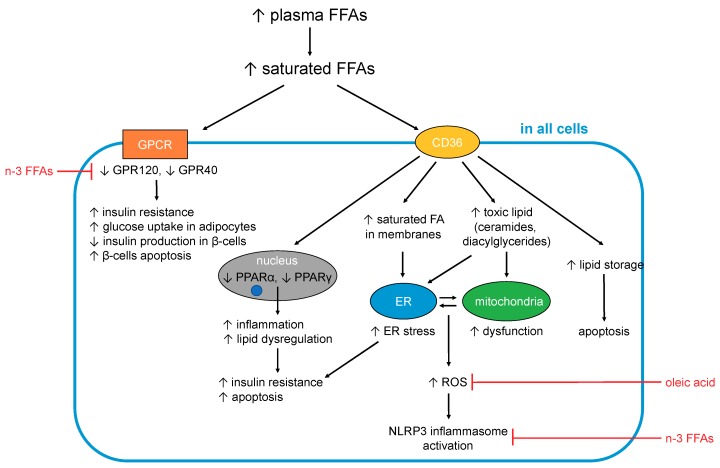
Schematic summarising the common effects of excess FFAs and saturated FFAs on metabolism and the different mechanisms through which insulin resistance can occur. CD36: Fatty acid translocase; GPCR (G protein-coupled receptor); ER (Endoplasmic reticulum); FA (fatty acids); FFA (free fatty acids); NLRP3 (NACHT, LRR and PYD domains-containing protein 3); PPAR (peroxisome proliferator-activated receptors); ROS (reactive oxygen species). GPCR and PPAR receptors have been introduced in Section 2.2.

In turn, some of the complications associated with T2DM are consequences of dysregulated FFA metabolism. In particular, adults with T2DM are two to three times more likely to develop cardiovascular disease compared to adults without diabetes, and nearly twice as likely to die from heart disease or a stroke [111,112]. In total, one third of adults with diabetes in the UK die from some form of cardiovascular disease compared to a quarter of adults in the general population [111]. Elevated FFAs may contribute to these outcomes in several ways. The effects caused by excess (saturated) FFAs on cells are summarised in Figure 1.

### 5.1. FFA-Induced Changes Occuring in All Cells and Tissues

#### 5.1.1. Generation of Toxic Lipids and Lipotoxicity

An excess of long-chain saturated (F)FAs in cells and tissues is associated with lipotoxicity [107]. When FFAs enter cells, most of them need to either be converted to TAGs for storage or to undergo β-oxidation to be used as fuel by mitochondria. However, when an excess of FFAs is present (especially of saturated FFAs), the cell becomes overwhelmed and full conversion to TAGs or complete β-oxidation becomes impossible, resulting in the generation of toxic lipids. Those toxic lipids include diacylglycerides and ceramide, which results from esterification of sphingosine with long chain saturated FFAs (in particular palmitic acid, but also stearic, arachidic, and linoceric acids (C24:0) but not shorter chain saturated FFAs (e.g., lauric (C12:0) and myristic acids (C14:0)) or unsaturated FFAs [113,114,115,116,117,118,119]. The build-up of those toxic lipids contributes to endoplasmic reticulum (ER) stress, mitochondrial dysfunction and the generation of reactive oxygen species (ROS), which together result in inflammation, insulin resistance and apoptosis (notably in adipocytes, β-cells and skeletal muscle cells) [113,114,115,116,117,118,119]. Several unsaturated FFAs including OA [120] and PUFAs [121] have been shown to counteract SFA-induced lipotoxicity. Although much remains to be elucidated, their ability to promote both TAG formation and β-oxidation may partially account for these observations [122].

#### 5.1.2. FFAs and Inflammation

Low-grade chronic inflammation constitutes a component of the aetiology and symptoms of T2DM. Indeed, the term “metaflammation” has been coined to describe this state of chronic metabolic inflammation [123], which—besides having many other deleterious effects—is the major underlying cause of insulin resistance [113]. Essentially, inflammatory signalling interferes with insulin signalling, with TNFα and several stress-related kinases (IκB kinase β (IKKβ), c-Jun N-terminal kinase (JNK)) being major mediators of insulin resistance [124]. The IKK complex inhibits NF-κB, the nuclear factor that stimulates secretion of inflammatory cytokines like IL-1, IL-6 (which are also known to induce insulin resistance) and TNFα. FFAs impact on inflammation in multiple tissues and organs via these and other pathways [108], briefly summarised here.

Different fatty acids can be metabolised into different pro- and anti-inflammatory signalling molecules [108], and so the amount and type of FFAs found in the blood (as a proxy for organismal FFA status) may have considerable bearing on signalling events that stimulate or alleviate inflammation. In particular, some n-6 FFAs (first and foremost ARA) are precursors to pro-inflammatory molecules (primarily prostaglandins), while n-3 FFAs (such as the n-3 PUFAs EPA and DHA) are precursors of resolvins that are anti-inflammatory [113]. However, it should be acknowledged that the n-6 FFAs LA and ARA are also precursors to anti-inflammatory agents [79].

Inflammation is one of the consequences of lipotoxicity (5.1.1), with SFAs acting as major inducers of inflammation through several mechanisms. One of these mechanisms involves the activation of toll-like receptor-4 (TLR-4) [125,126], which normally responds to pathogen-derived lipopolysaccharides [126,127]. TLR-4-mediated signalling activates the production of pro-inflammatory cytokines (including interleukins 1β and 6, TNFα) and expression of cyclooxygenase-2, a critical enzyme for the conversion of ARA into pro-inflammatory prostaglandins. TLR-4 is more activated in T2DM cases; together with elevated SFAs, its stimulation promotes inflammation. Conversely, n-3 FFAs can suppress this pathway through the inhibition of TLR-4 dimerisation and reduced incorporation into lipid rafts [125,128,129,130]. Little is known about the effect of MUFAs or other PUFAs. One cell culture study found that OA did not, in contrast to palmitic acid, activate TLR-4 in macrophages [131], and in rats, only a high n-3:n-6 PUFA ratio reduced TLR-4 expression. 

Furthermore, SFAs activate the NLRP3 (NACHT, LRR and PYD domains-containing protein 3) inflammasome multi-protein complex [132], which under normal conditions mediates processing and secretion of interleukin-1β. The latter cytokine is an important mediator of the inflammatory response (e.g., in response to infection with pathogens). The NLRP3 inflammasome assembles on mitochondria, and senses general disturbance in redox and ion (Ca^2+^) balance. Besides these triggers, it can also be activated by mitochondrial DNA and cardiolipin, a membrane lipid present in mitochondria and bacteria. Thus, one of the triggers for NLRP3 inflammasome assembly is severe mitochondrial dysfunction, which can be a consequence of disturbed FFA β-oxidation. While this mechanism is known to occur in different tissues, NLRP3 inflammasome activation in pancreatic β-cells and islet-infiltrating macrophages is of particular relevance here, as it was found to lead to impaired insulin secretion and increased apoptosis [132]. Palmitic acid but not OA is able to activate the NLRP3 inflammasome, while several n-3 [132,133] and n-6 [133] FFAs inhibit NLRP3 inflammasome activation.

Further SFA-related processes occurring in mitochondria have also been proposed to contribute to inflammation in several ways. Either increased or erratic β-oxidation of FFAs is thought to generate reactive oxygen species (ROS) and ultimately to cause oxidative stress. ROS activate several stress-related kinases (JNK and IKKβ) that are associated with inflammation [113]. Furthermore, ROS cause ER stress, which is also associated with inflammation [132]. SFAs can cause ER stress in a variety of cell types [132], with the generation of ROS just one of several mechanisms by which this may occur. ER stress is associated with dysregulation of Ca^2+^ signalling; palmitic acid has been shown to induce this alongside oxidative stress [24]. Both mitochondrial dysfunction and ER stress can eventually lead to apoptosis. These latter processes are important at later stages of (poorly managed) T2DM and contribute to the loss of pancreatic β-cells and concomitant decrease in insulin secretion, as well as to cardiovascular complications. 

Regarding differential effects of different FFAs, the cellular and molecular biological information detailed above is predominantly derived from in vitro and animal studies. As can be seen from the discussion in Section 3, consumption or administration of particular types of (F)FAs may not necessarily exert the effects expected based on these data [134]. Nonetheless, exploiting these insights for “nutritional modulation” for reducing metabolic inflammation is actively being investigated [135]. Indeed, some studies in humans have observed correlations between inflammatory markers and specific FFAs. For example, in a prospective study involving women with or without gestational diabetes [136], inflammatory markers were inversely correlated with palmitoleic, oleic, linolenic, and myristic acids, whilst positive associations were found with palmitic, stearic, arachidonic, dihomo-γ-linolenic, and docosahexaenoic acids.

#### 5.1.3. Effects on Cellular Membranes

Finally, an excess of saturated FFAs also impacts the fatty acid composition of cellular membranes. This directly affects cell functioning through changes in membrane fluidity and permeability to ions and molecules, as well as the incorporation of insulin receptors into the membrane [137]. Indeed, a direct association has been shown between an increased proportion of SFAs in membrane phospholipids and T2DM, with the higher membrane rigidity caused by the increased presence of saturated chains hypothesised to lead to impaired insulin signalling [137,138]. FFA elongation, desaturation and esterification occur at the ER membrane [132], and when SFAs are in excess, morphology and functioning of the ER may become altered [139], leading to ER stress. 

In summary, an excess of SFAs exerts multiple deleterious effects on cellular functions. This is mediated by differential activation of GPCRs (GPR40 and GPR120) and PPARs, less efficient conversion to TAG and resulting generation of toxic lipids (diacylglycerides and ceramide), generation of ROS and excessive incorporation into the membranes of cells and subcellular compartments. Several of these processes are associated with inflammatory signalling cascades and/or insulin signalling. 

### 5.2. FFA-Induced Changes in Specific Organs and Tissues

**Figure 2:**
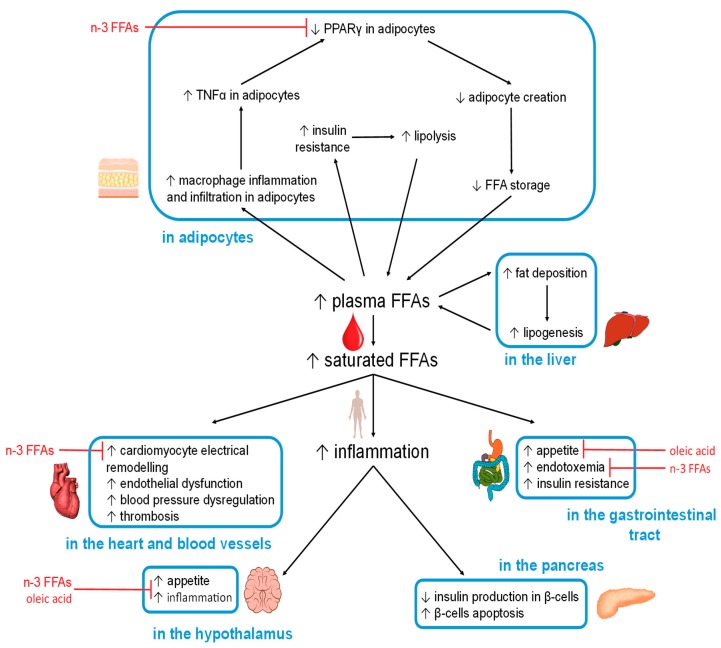
Schematic summarizing the organ-specific effects of excess FFAs and saturated FFAs on metabolism and the different mechanisms through which insulin resistance can occur. FA (fatty acids); FFA (free fatty acids); PPAR (peroxisome proliferator-activated receptors); TNFα (tumour necrosis factor α).

The organ-specific effects caused by excess (saturated) FFAs, and observed beneficial effects of other FFAs, are summarised in Figure 2.

#### 5.2.1. Hypothalamus

Excess FFA intake is known to negatively affect the regulation of satiety in the hypothalamus. The accumulation of toxic lipids caused by saturated FFAs results in neuronal inflammation through TLR-4 activation [113,140,141]. This inflammation then inhibits the actions of leptin and insulin, leading to increased appetite [142]. In addition, endothelial CD36(FAT) in capillary vessels (at the blood-brain barrier) [143] can detect elevated levels of certain plasma FFAs [113]. If the plasma FFA pool is high in palmitic acid, the hypothalamic-pituitary-adrenal axis is stimulated to release more cortisol, a process that contributes to insulin resistance [144]. This is because palmitic acid is the main product of de novo lipid synthesis in the liver, a mechanism activated when glucose is in excess. On the other hand, if the plasma FFA pool is high in the MUFA OA, then the production of the hunger-inducing neuropeptide Y hormone will be reduced [113]. Moreover, ALA and OA were able to revert high-fat induced hypothalamic inflammation in a mouse model [145]. 

#### 5.2.2. Gastrointestinal Tract

The dysregulation of hypothalamic functioning therefore increases food intake, including FFAs that then act within the gastrointestinal tract. In the mouth, fatty acid receptors such as GPR40 and GPR120, as well as fatty acid transporters/sensors such as CD36, interact directly with FFAs [22]. CD36 in particular binds OA and participates in its conversion into oleylethanolamide, a molecule that regulates satiety as well as β-oxidation [146,147]. Specific cells present in the gastrointestinal tract (enteroendocrine cells) produce hormones in response to specific nutrients that include fat [148]. Among those is cholecystokinin (CCK), which is secreted by the I-cells in the proximal small intestine. CCK suppresses hunger by interacting with the hypothalamus via the vagus nerve and reduces gluconeogenesis in the liver. However, both of these actions are perturbed when the hypothalamus is inflamed [149,150,151,152,153].

Apart from direct effects of (F)FAs from the diet, it is now also becoming increasingly clear how diet affects the gastrointestinal microbiome, and how this may contribute to predisposing patients to obesity, metabolic syndrome and T2DM [154,155,156,157]. The fact that T2DM patients exhibit disturbances in their gut microbiomes is in keeping with this. Studying the links between diet, microbiome, and diseases enables the use of diet as a therapeutic approach; a recent interventional study has indicated how a high-fibre, polyphenol-rich and vegetable-protein diet affects the gut microbiome, plasma FFAs, and glucose tolerance [158].

Importantly, FAs are one major component in these interactions—both in terms of cause and effect. The type of fat consumed affects the composition of the gut microbiome, which in turn has critical effects on the end products of bacterial fermentation in the gut [159]. A decrease in the production of short-chain FFAs (acetic, propionic and butyric acid) from dietary fibre is a risk factor for T2DM [160]. Short-chain FFAs are used as an energy source, as metabolites and signalling agents, and have a range of beneficial effects (for example, propionic acid improves insulin sensitivity [161]).

A diet rich in saturated fat may cause an imbalance in the gastrointestinal microbiome [162], which can also increase circulating bacterial endotoxin, primarily lipopolysaccharides (LPS) [108,158,162]. This may contribute to transient or chronic inflammation in obese and T2DM patients [159], thought to be mediated through TLR-4 in adipocytes [162]. Causative links between gut microbial endotoxin and inflammation have also been suggested as contributing factor to insulin resistance [163,164] and glucose intolerance [158]. Notably, dietary changes can influence this interplay positively [158].

#### 5.2.3. Adipocytes

When FFA intake or de-novo synthesis is increased, the resultant excess FFAs are required to be stored in the form of TAGs in adipocytes, the only cells in the body which can store large amounts of fat (relatively) safely. However, increased fat storage beyond a certain threshold can cause the adipocytes to expand too much, causing hypoxia, which results in inflammation (in addition to the previously mentioned inflammatory pathways). This is reflected by an increase in macrophage infiltration in adipose tissue, where macrophages can constitute up to 50% of the total mass of the adipose tissue in obese individuals (compared to 10% in lean individuals). Further to this, macrophages are more likely to adopt a pro-inflammatory state in obese individuals [165,166]. Indeed, adipose tissue is thought of as the major inflammatory organ that mediates obesity-induced inflammation [167]. Amongst others, adipose tissue releases inflammatory cytokines such as TNFα, which is associated with increased insulin resistance and increased lipolysis [168,169]. TNFα inhibits PPARγ, the activity of which is necessary for the generation of new adipocytes from stem cells present in adipose tissue [170,171]. This action is in opposition with anti-inflammatory nutrients such as n-3 FFAs (EPA and DHA) that increase PPARγ activity and adipocyte production [172]. There are further beneficial effects associated with n-3 FFAs through their binding to GPR120 in adipocytes, resulting in glucose transporter type-4 (GLUT-4) translocation, which enhances glucose uptake by adipocytes (GLUT-4 membrane concentration is reduced in T2DM) [23,173]. 

In the absence of new adipocytes, caused by decreased PPARγ activation, the adipocytes that are already present will continue to expand in size until they die, with the resultant cellular debris triggering further inflammation [174]. This prevents adipocyte-storage of excess dietary FFAs, leading to increased FFAs in plasma and tissues. Further increases in plasma FFAs can be caused by hyperinsulinemia (often associated with insulin resistance), which activates lipoprotein lipase at the surface of adipocytes. This results in the hydrolysis of TAGs within lipoproteins to release FFAs. At the same time, hyperinsulinemia also increases synthesis of CD36, which acts to carry FFAs into the adipocytes for deposition [175,176,177,178]. Furthermore, T2DM can also lead to hypoinsulinemia and an increase in plasma FFAs, as insulin reduces the activity of hormone-sensitive lipase, a protein required for lipolysis [179]. This means that when insulin levels fall, lipolysis is increased. Thus, FFA metabolism in T2DM is characterised by greater FFA flux both into and out of adipocytes [113,180]. The implications are that increased plasma FFA concentrations lead to insulin resistance which then itself, through increased lipolysis, results in a further increase in plasma FFA concentration; this constitutes a vicious cycle affecting FFAs in T2DM. As a result of adipocyte dysregulation, excess FFAs are released into the bloodstream and are taken up by other organs, such as the liver, pancreas, heart and skeletal muscles, where fat then accumulates. This induces lipotoxicity and impacts upon cellular functions. The type of FFAs present affects how those processes progress. Notably, palmitoleic acid (C16:1n-7) secreted by adipose tissue acts as a lipokine and can prevent excessive fat accumulation in the liver [181].

#### 5.2.4. Liver

The liver is a critical hub for energy metabolism, and the major location for de-novo fatty acid synthesis (from carbohydrates) and gluconeogenesis (glucose production, including from fatty acids). When plasma FFA levels are high, some of the excess is taken up by the liver, but its storage capacity is limited. This often results in the build-up of fat deposits, a major factor in the development of non-alcoholic fatty liver disease [182], a condition shared by 69% of the individuals with T2DM in the USA and up to 90% of individuals with both T2DM and severe obesity [183]. In non-alcoholic fatty liver disease the ability of the liver to suppress glucose production when present in sufficient quantity is reduced [184].

#### 5.2.5. Pancreas

The pancreas is another organ which is critically affected by chronic elevation in plasma FFAs. The β-cells of the pancreas are very sensitive to inflammation and this can compromise insulin secretion and ultimately cause the destruction of the β-cells [185]. Pancreatic β-cells are affected by all major mechanisms mentioned in Section 5.1, including SFA-induced lipotoxicity [117,186], pro-inflammatory FFA-derived molecules such as 12-hydroxyeicosatetraenoate [185], ROS production and oxidative stress [114], and ER stress [116]. In vitro cell culture experiments have demonstrated differential effects of different FFAs, where palmitic acid but not OA exposure caused β-cell death [115].

#### 5.2.6. Heart

FFAs and their esters are the major source of fuel for the heart muscle. However, an excess of plasma FFAs has profound effects on the heart causing an enhanced susceptibility to oxidative stress and ischemic damage [12]. The formation of toxic lipids from excess FFAs can activate protein kinase C that then impairs intracellular Ca^2+^ handling and cardiac contractibility, which promotes cardiac fibrosis and hypertrophy [12,187]. Also here, different types of FFAs exert opposing effects: saturated FFAs can induce structural and electrical remodelling of atrial myocytes (possibly by activation of TLRs on macrophages that then infiltrate or couple with myocytes). An excess of saturated FFAs may also cause molecular remodelling of cardiac ion channels through TLR-4, NF-κB and IL-6-dependent [188] inflammatory mechanisms, leading to electrophysiological remodelling and sustained and fatal arrhythmias [5,188]. In contrast, n-3 PUFAs have anti-arrhythmic and cardio-protective effects [188].

#### 5.2.7. Blood Vessels

Endothelial dysfunction, a precursor to atherosclerosis and cardiovascular diseases, is induced through different mechanisms by excess FFAs in plasma [189]. The activation of the NF-κB inflammation pathway by saturated but not polyunsaturated FFAs results in increased endothelial superoxide production and reduced nitric oxide production, while NLRP3 inflammasome activation potentially leads to an increase in endothelial permeability [189]. Nitric oxide production is also reduced by an excess of FFA through dysregulation of Ca^2+^ and insulin signalling [189]. An excess of FFAs can influence the renin-angiotensin system, which can lead to dysregulation of arterial blood pressure [189]. Palmitic acid contributes to the apoptosis of endothelial cells via the p38 and JNK/MAPK pathways and can upregulate MEG3 RNA production, an important molecule required for endothelial cell differentiation [189].

Excess FFAs in the blood also lead to FFA adherence to the endothelial walls of the vessels, and their subsequent accumulation contributes to the formation of atherosclerotic plaques which can partially block the vessel and whose rupture can trigger thrombosis and embolism [189]. One player in this complex process is the FA translocase CD36 on macrophages and platelets [25], which in the latter cells leads to a hyper-active state that promotes coagulation and hence thrombosis.

Furthermore, the fact that the major plasma FFA carrier serum albumin is also the major zinc transporter in plasma has critical implications, not limited to thrombosis and atherosclerosis [190]. The binding of long-chain FFAs including palmitic acid triggers a conformational change in the protein that affects its ability to bind zinc. By this allosteric mechanism, elevated FFAs result in dysregulation of plasma zinc handling and disturbed circulatory zinc transportation. The zinc ion (Zn^2+^) is an important signalling agent; amongst many other effects, it is an important regulator of coagulation [191,192]. It is thought that displaced Zn^2+^ binds to coagulatory proteins, influencing their activity, leading to a pro-thrombotic effect [190,191,192,193]. In addition, Zn^2+^ is also essential for insulin storage in β-cells. T2DM is associated with zinc deficiency [194], which could partially be explained by impaired zinc transport in the blood. In turn, zinc deficiency is known to negatively affect insulin storage and secretion [195]. Finally, elevated plasma FFAs have been shown to alter fibrin clot parameters in a purified system and may have a similar effect in plasma [196]. The effects of stearic acid and OA were not the same, with stearic acid increasing the diameter of fibrin fibres, while OA reduced it. Both FFAs reduced the mechanical stability of the clot (decreased rigidity, higher deformability and decreased internal resistance to shear stress) and increased clotting time [196].

#### 5.2.8. Skeletal Muscle

Skeletal muscle is the main location for glucose and FFA utilisation. The higher flux of TAG and FFAs typical in T2DM leads to increased uptake of FFAs into myocytes [122]. Excess is stored as intramyocellular TAG; this is in principle a normal physiological process, but there are healthy limits that depend on how much the muscles are used. Most consequences of increased cellular FFA accumulation described in Section 5.1 apply to myocytes, including the production of diacylglycerides and ceramide and ensuing lipotoxicity, the development of inflammation and insulin resistance, and eventually cell damage. Like the heart muscle, skeletal myocytes are rich in mitochondria. They adapt to increased FFA flux by increased β-oxidation, but due to overwhelming of the Krebs cycle and the electron-transport chain, this seems to be accompanied by incomplete oxidation, accumulation of acylcarnitine, increased ROS production, and resultant mitochondrial stress [122].

In summary, elevated plasma FFAs are involved in promoting the metabolic changes associated with insulin resistance in multiple cell types and T2DM, which in turn leads to increased plasma FFA levels and flux into cells. As FFA interactions with plasma molecules, as well as their effects on cells, differ depending on their chain length and saturation status, it is important to identify which specific FFAs have altered plasma concentrations in T2DM. In addition, an important treatment option in T2DM is the use of lipid-lowering drugs. While the primary targets of those drugs are generally other forms of lipids (e.g., cholesterol), part of their beneficial effect stems from the fact that they can lower plasma FFA levels. Thus, it is interesting to consider how those drugs affect particular FFAs. 

## 6. Effects of Interventions Using Lipid-Lowering Drugs

### 6.1. Changes in Total Plasma FFA Concentration

It is clear that the elevated plasma FFA levels associated with T2DM can lead to numerous pathologies; it is thus clinically important to control their concentrations. Besides other medications such as metformin (vide infra), T2DM is pharmacologically managed using lipid-lowering drugs [197]. Many different strategies have been used to design those drugs, as reviewed by Barter et al. [198], with much of the focus given to the ability of such drugs to control the plasma levels of cholesterol, LDL and HDL rather than levels of FFAs. However, it is established that several of those drugs can also affect plasma FFA concentrations through different mechanisms. Table 3 lists clinical trials and studies performed on human patients in which the effect of specific drugs on plasma FFA levels have been measured. 

Metformin was one of the first drugs used to treat T2DM. It is not directly targeted to lower lipid levels but rather blood glucose. It exerts its action through inhibition of gluconeogenesis in the liver, increasing glucose sensitivity and decreasing glucose intestinal absorption [199]. Metformin affects lipids through activation of 5’ AMP-activated protein kinase (AMPK) resulting in an increase in β-oxidation and the inhibition of lipogenesis [199]. Studies on the effects of metformin administration from 15 days up to 6 months have been carried out [200,201,202,203,204,205]. Most studies (on healthy subjects, subjects with T2DM, subjects with hyperlipidaemia and in relatives of T2DM subjects with impaired glucose tolerance) suggest that the drug has no effect on total plasma FFA concentrations [200,202,203,205], whilst two studies (on healthy subjects and on subjects with reduced glucose tolerance) concluded that metformin can reduce plasma FFA concentration in T2DM [201,204].

Statins are currently the main class of drugs used to lower lipids in T2DM subjects. They inhibit HMG-CoA (3-hydroxy-3-methyl-glutaryl-coenzyme A) reductase, an enzyme involved in the synthesis of cholesterol in cells [198]. Inhibition of HMG-CoA increases the expression of LDL receptors on the cell surface, causing increased uptake of LDL by cells and consequently reducing plasma LDL concentration. A recent meta-analysis examining the effects of long-term administration of statins on total plasma FFA concentration in individuals with T2DM, metabolic syndrome or dyslipidaemia has shown that it can also reduce the total plasma FFA concentration [206]. Fibrates can be used as an alternative drug to statins [207,208]. They activate PPARα, resulting in an increase in the oxidation of FFAs in the liver and an inhibition of de novo FFA synthesis, thus reducing the availability of FFAs for triglyceride synthesis and so reducing TAG levels [207]. The effects of fibrates on total plasma FFA concentrations have been extensively investigated [209,210,211,212,213,214,215,216,217,218,219,220,221,222,223]. All studies have found unchanged or reduced total FFAs concentrations in a variety of populations (healthy subjects and subjects with T2DM, hypolipoproteinaemia, hyperinsulinemia, hypertriglyceridemia, glucose intolerance or metabolic syndrome), with the exception of one study that reported an increase in total plasma FFA concentration in subjects with hypertriglyceridemia and glucose intolerance [218].

Ezetimibe inhibits cholesterol absorption through the inhibition of Niemann-Pick C1-Like 1 protein in the intestine [198]. The effect of long-term administration of ezetimibe (6 months) has been investigated in patients with T2DM and in patients with hypercholesterolemia (with and without insulin resistance) [224,225]. The drug was found to reduce the total plasma FFA concentration but only in subjects with T2DM or with hypocholesteraemia and insulin resistance. In hypocholesteraemic subjects without insulin resistance the FFA levels after treatment were unchanged.

Niacin or nicotinic acid is a form of vitamin B3. It inhibits lipolysis via activation of GPR109A [226]. In several one-day trials niacin has been shown to reduce the postprandial concentration of total plasma FFA in healthy and obese subjects [227,228,229,230], although its effects are more complex. In healthy subjects, total FFA concentrations are reduced immediately after niacin administration and a meal, but a rebound occurs around an hour later [231]. Once niacin is administered in the long-term, this rebound negates the reduction of total FFA concentrations in the fasting state after one to two weeks [232] and can lead to an increase after one month of treatment [231]. The longer-term effects of niacin administration have yet to be investigated in subjects with T2DM. As niacin can provoke adverse secondary effects in patients, especially flushing, other drugs have been developed to replicate the effects of niacin but without these secondary effects. Those include GSK256073, a hydroxy-carboxylic acid receptor-2 agonist and both MK-1903 and SCH900271, which are GPR109A agonists [233,234]. These drugs have been shown to reduce total plasma FFA concentrations in the short-term, but lead to unchanged or even elevated concentrations in the long-term.

**Table 3:** List of studies having measured the total FFA concentrations in the blood from subjects treated with particular lipid-lowering drugs and characteristics of the populations studied. Studies shorter than 1 week, not comparing the effect of the drugs to a placebo or done on population with diseases other than T2DM, glucose intolerance, insulin resistance, obesity, metabolic syndrome or dyslipidaemia were excluded, while studies on healthy subjects were included).

Drug Type	Reference	Subjects	Drug Treatment	Effect
Metformin	Pentikäinen et al., 1990 [200]	24 non-diabetic subjects with hyperlipidaemia	Randomised, double blind, placebo-controlled, crossover study. Metformin 1 g/day or 2 g/day or placebo for 9 weeks.	Unchanged fasting total FFA levels.
	Landin et al., 1994 [201]	18 healthy subjects	Randomised, double-blind, placebo-controlled, triple crossover study. Metformin 850 mg twice daily or metoprolol 100 mg/day for 18 weeks.	Reduced fasting total FFA levels.
	Lehtovirta et al., 2001 [202]	40 first-degree relatives of T2DM patients with impaired glucose tolerance	Block-randomised, double-blind, placebo-controlled, parallel group study. Metformin 500 mg twice daily for 6 months.	Unchanged fasting total FFA levels.
	Fruehwald-Schultes et al., 2002 [203]	15 healthy men	Double-blind, placebo-controlled, crossover study. Metformin 850 mg twice daily for 15 days.	Unchanged fasting total FFA levels.
	Krysiak et al., 2012 [204]	58 subjects with impaired fasting glucose	Randomised, placebo-controlled, parallel group study. Simvastatin 40 mg/day + either metformin 1 g thrice daily for 3 months.	Reduced fasting total FFA levels.
	Gormsen et al., 2018 [205]	24 T2DM subjects, 12 healthy subjects	Randomised, placebo-controlled, parallel-group trial. Metformin 1 g twice daily for 3 months.	Unchanged fasting total FFA levels.
Statins	Sahebkar et al., 2016 [206]	Subjects with T2DM, metabolic syndrome and dyslipidaemia	Meta-analysis. Atorvastatin or simvastatin, less than or more than 12 weeks	Reduced total FFA levels.
Fibrates	Fenderson et al., 1974 [209]	4 healthy subjects, 27 subjects with hypolipoproteinaemia	Controlled, parallel group study. Clofibrate 2 g/day for 21 days	Reduced fasting total plasma FFA levels and reduced levels during oral glucose tolerance test in hypolipoproteinaemia subjects but not in healthy subjects.
	Calvert et al., 1980 [210]	22 T2DM subjects	Randomised, double-blind, placebo-controlled, crossover study. Clofibrate 1 g twice daily for 12 weeks.	Reduced fasting and 8 h-average total plasma FFA levels.
	Jones et al., 1990 [211]	36 T2DM subjects	Randomised, double-blind, placebo-controlled, parallel group study. Bezafibrate 200 mg thrice daily for 3 months.	Reduced fasting and postprandial total plasma FFA levels.
	Alberti et al., 1990 [212]	20 T2DM subjects	Randomised, double-blind, placebo-controlled, parallel group study. Bezafibrate 200 mg thrice daily for 3 months.	Reduced fasting total plasma FFA levels.
	Vuorinen-Markkola et al., 1993 [213]	20 T2DM subjects	Randomised, double-blind, placebo-controlled, parallel group study. Gemfibrozil 1200 mg/day for 12 weeks.	Unchanged 24 h-average total plasma FFA levels.
	Sane et al., 1995 [214]	20 subjects with hyperinsulinemia and hypertriglyceridemia	Randomised, double-blind, placebo-controlled, parallel group study. Gemfibrozil 1200 mg/day for 12 weeks.	Unchanged 24 h-average total plasma FFA levels and during insulin infusion.
	Avogaro et al., 1995 [215]	18 subjects with hypertriglyceridemia, 11 with T2DM, 7 without T2DM	Randomised, single-blind, placebo-controlled, cross-over study. Gemfibrozil 600 mg twice daily for three months.	Reduced fasting total plasma FFA levels for both populations. Reduced postprandial total FFA levels for the T2DM group.
	Jeng et al., 1996 [216]	24 subjects with hypertriglyceridemia	Randomised, placebo-control, parallel group study. Gemfibrozil 600 mg twice daily for 3 months.	Reduced postprandial total plasma FFA levels.
	Avogaro et al., 1999 [217]	217 T2DM subjects	Randomised, double-bling, placebo-controlled, parallel group study. Gemfibrozil 600 mg twice daily for 20 weeks.	Unchanged fasting total plasma FFA levels.
	Mussoni et al., 2000 [218]	56 subjects with hypertriglyceridemia and glucose intolerance	Randomised, double-blind, placebo-controlled, parallel group study. Gemfibrozil 600 mg twice a day for 5 months.	Increased fasting total plasma FFA levels.
	Jonkers et al., 2001 [219]	17 subjects with hypertriglyceridemia	Randomised, double-blind, placebo-controlled, crossover study. Bezafibrate 400 mg/day for 6 weeks.	Reduced fasting total plasma FFA levels.
	Capell et al., 2003 [220]	11 subjects with hypertriglyceridemia	Randomised, double-blind, placebo-controlled, crossover study. Fenofibrates for 14 days.	Unchanged 24 h-average total plasma FFA levels but reduced levels after heparin infusion.
	Vega et al., 2003 [221]	13 men with metabolic syndrome	Randomised, placebo-controlled, crossover study. Fenofibrates 200 mg/day for 8 weeks.	Unchanged fasting total plasma FFA levels and during oral glucose tolerance test.
	Li et al., 2011 [222]	87 obese subjects with hyperinsulinemia but not diabetes, on metformin.	Randomised, double-blind, placebo-controlled, parallel group study. Fenofibrate 200 mg/day for 6 months.	Reduced fasting total plasma FFA levels.
	Matsuba et al., 2018 [223]	27 subjects with hypertriglyceridemia and insulin resistance	Randomised, double-blind, placebo-controlled, parallel group study. Pemafibrate 0.4 mg/day, twice daily for 12 weeks	Reduced fasting total plasma FFA levels.
Ezetimibe	Krysiak et al., 2014 [224]	39 subjects with hypercholesterolemia and 20 healthy controls	Controlled, parallel group study. Ezetimibe 10 mg/day for 90 days	Reduced fasting total FFA levels in insulin-resistant patients, unchanged in patients without insulin resistance but with hypercholesterolemia.
	Sugiyama et al., 2015 [225]	33 T2DM patients,	Randomised, open-label, controlled, parallel group study. Ezetimibe 10 mg/day for 6 months	Reduced fasting total FFA levels.
Nicotinic acid (niacin)	Kelly et al., 2000 [232]	7 healthy subjects	Randomised, double-blind, placebo-controlled, crossover study. Nicotinic acid 500 mg for 7 days, then 2 g/day for 7 days.	Unchanged fasting total FFA levels.
	Wang et al., 2000 [231]	5 healthy women	Single-blind, controlled, parallel group study. Increasing nicotinic acid doses over 1 month up to 1g or placebo.	After 1 month, on-significant elevated fasting total FFA levels, decreased upon taking niacin up to 1 h 30 afterwards, but large rebound from 3 to 6 h afterwards.
Niacin mimetic (hydroxy-carboxylic acid receptor 2 agonist)	Dobbins et al., 2015 [233]	94 T2DM patients	Randomised, double-blind, placebo-controlled, parallel group study. GSK256073, 5 or 25 mg twice daily or 10 or 50 mg once daily, for 12 weeks.	Reduced fasting total FFA levels at day 2 but less effective or no effect at week 6.
Niacin mimetic (GPR109A agonist)	Lauring et al., 2012 [234]	Subjects with mixed dyslipidaemia, 162 for MK-1903 study, 69 for SCH900271 study	Randomised, double-blind, placebo-controlled, parallel group study. MK-1903 150 mg Q8h doses for 4 weeks. Randomised, partially blind, placebo-controlled, parallel-group study. SCH900271 10 mg for 29 days.	MK-1903 reduced fasting total FFA levels at day 1. At day 28 both drugs reduced fasting total FFA levels upon drug intake, but intake induced an immediate rebound which results in elevated 8h-average total FFA levels.
Bile acid sequestrants	Vega et al., 2011 [235]	20 men with metabolic syndrome	Randomised double-blind, placebo-controlled crossover study. Colesevelam 1.875 g twice daily for 8 weeks.	Reduced fasting total FFA levels but increased postprandial total FFA levels.
Thiazolidine-diones	Chaiken et al., 1995 [236]	19 obese T2DM subjects	Randomised, double-blind placebo-controlled, parallel group study. Darglitazone 25 mg/day for 14 days.	Reduced 24 h-average total plasma FFA levels.
	Buysschaert et al., 1999 [237]	259 T2DM subjects	Randomized, double-dummy, placebo-controlled, parallel-group study. Troglitazone 100 or 200 mg/day for 16 weeks.	Reduced fasting total plasma FFA levels.
	Raskin et al., 2000 [238]	303 T2DM subjects	Randomised, double-blind, placebo-controlled, parallel group study. Rosiglitazone, 0, 2, 4 or 6 mg twice daily for 2 weeks.	Reduced fasting total plasma FFA levels.
	Miyazaki et al., 2001 [239]	29 T2DM subjects	Randomised double-blind, placebo-controlled, parallel group study. Rosiglitazone 8 mg/day for 12 weeks.	Reduced fasting total plasma FFA levels and reduced level during oral glucose tolerance test.
	Kerenyi et al., 2004 [240]	340 T2DM subjects	Randomised double-blind, placebo-controlled, parallel group study. Rosiglitazone 4 mg twice daily for 26 weeks.	Reduced fasting total plasma FFA levels.
	James et al., 2005 [241]	30 obese and insulin resistant men	Randomised, placebo-controlled, parallel-group study. Metformin 1 g twice daily or rosiglitazone 4 mg twice daily for 8 weeks	Unchanged fasting total plasma FFA levels.
	Tan et al., 2005 [242]	24 T2DM subjects	Randomised, double-blind, placebo-controlled, cross-over study. Rosiglitazone 4 mg twice daily for 12 weeks.	Unchanged fasting total plasma FFA levels, reduced postprandial levels.
	Al Majali et al., 2006 [144]	22 T2DM patients, 10 healthy controls	Randomised, double-blind, placebo-controlled, parallel group study. Pioglitazone 45 mg/day or glibenclamide 5 mg/day	Unchanged fasting or postprandial total plasma FFA levels.
	Samaha et al., 2006 [243]	57 nondiabetic subjects with metabolic syndrome	Randomised, double-blinded, placebo-controlled, parallel group study. Rosiglitazone 8 mg/day for 12 weeks.	Unchanged fasting total plasma FFA levels.
	Mittermayer et al., 2007 [244]	16 healthy men	Randomised, double-blind, placebo-controlled, parallel-group study. Rosiglitazone 8 mg/day for 21 days.	Reduced total plasma FFA levels before and 5 h after lipid infusion.
	Miyazaki et al., 2007 [245]	29 T2DM subjects	Randomised, double-blind, placebo-controlled, parallel group study. Rosiglitazone 8 mg/day for 12 weeks.	Reduced fasting total plasma FFA levels and reduced levels during oral glucose tolerance test.
	Krzyzanowska et al., 2007 [246]	16 healthy men	Randomised, double-blind, placebo-controlled parallel-group study. Rosiglitazone 8 mg/day for 21 days.	Reduced total plasma FFA levels before and 5 h after lipid infusion.
	Abbasi et al., 2008 [247]	37 overweight, nondiabetic, insulin resistant subjects	Randomised, controlled, parallel group study. Fenofibrate 160 mg/day for 12 weeks or rosiglitazone 4 mg once daily for 4 weeks, then 4 mg twice daily for 8 weeks.	Reduced daylong (8 h-average) total plasma FFA levels.
	Punthakee et al., 2014 [248]	190 subjects with impaired fasting glucose or impaired glucose tolerance	Randomised, double-blind, placebo controlled, parallel group study. Rosiglitazone 4mg/day for the first 2 month then 8 mg/day for 3.5 year	Unchanged fasting total plasma FFA levels.
	Kim et al., 2014 [249]	173 subjects with T2DM	Randomised, double-blind, placebo-controlled, parallel-group study. Lobeglitazone 0.5 mg/day for 24 weeks.	Reduced fasting total plasma FFA levels.
n-3 fatty acid	Farsi et al., 2014 [250]	44 T2DM subjects	Randomised, double-blind, controlled, parallel group study. n-3 soft gels 4 g/day for 10 weeks.	Reduced fasting total plasma FFA levels.
ETC-1002	Thompson et al., 2015 [251]	56 hypercholesterolemia subjects with statins intolerance	Randomised, double-blind, placebo-controlled, parallel group study. ETC-1002 60 mg/day increased every 2 weeks to 120 mg/day, 180 mg/day and 240 mg/day for a total of 8 weeks.	Unchanged fasting total FFA levels.
Combinations of drugs	Gómez-Perez et al., 2002 [252]	116 T2DM subjects	Randomised, double-blind, placebo-controlled, parallel group study. Metformin 2.5 g/day and placebo, metformin 2.5 g/day and rosiglitazone 2 mg twice daily, or metformin 2.5 g/day and rosiglitazone 4 mg twice daily for 26 weeks.	Reduced fasting total FFA levels.
	Wagner et al., 2005 [253]	12 healthy subjects	Randomised, placebo-controlled, incomplete-block, 3-period crossover study. Fenofibrate 201 mg/day, rosiglitazone 4 mg twice daily, or combined fenofibrate 201 mg/day and rosiglitazone 4 mg twice daily.	Reduced fasting total FFA levels.
	Boden et al., 2007 [254]	13 T2DM subjects	Single-blind placebo-controlled, parallel group study. Rosiglitazone 8 mg/day, fenofibrate 160 mg/day or Rosiglitazone 8 mg/day and fenofibrate 160 mg/day for 2 months.	Reduced daily-average total FFA levels.
	Plat et al., 2009 [255]	36 subjects with metabolic syndrome	Randomised, double-blind, placebo-controlled, parallel group study. Simvastatin 10 mg/day, plant stanols 2g/day, or simvastatin 10 mg/day and plant stanols 2 g/day for 9 weeks	Unchanged fasting total FFA levels.
	Bays et al., 2011 [256]	183 subjects with dyslipidaemia	Randomised, double-blind, placebo-controlled, parallel group study. MBX-8025 50 mg/day; MBX-8025 100 mg/day; atorvastatin 20 mg/day; MBX-8025 50 mg/day and atorvastatin 20 mg/day; or MBX-8025 100 mg/day and atorvastatin 20 mg/day for 8 weeks.	Reduced fasting total plasma FFA levels for MBX-8025 50 or 100 mg/day and for MBX-8025 50 mg/day and atorvastatin 20 mg/day. Unchanged levels for atorvastatin alone or with MBX-8025 100 mg/day.
	Krysiak et al., 2014 [257]	65 subjects with hypercholesterolemia	Randomised, not blinded, placebo-controlled, parallel group study. Simvastatin 40 mg/day; ezetimibe 10 mg/day; or simvastatin 40 mg/day and ezetimibe 10 mg/day for 12 weeks.	Reduced fasting total plasma FFA levels.
	Hwang et al., 2019 [258]	36 T2DM subjects	Randomised, open-label, active-control, parallel group study. Rosuvastatin 20 mg/day or rosuvastatin 5 mg/day and ezetimibe 10 mg/day for 6 weeks.	Reduced fasting total plasma FFA levels.

Bile acid sequestrants (e.g., cholestyramine, colestipol, and colesevelam) bind bile acid in the intestine to prevent its reabsorption [198]. Synthesis of new bile acid requires cholesterol, leading to the reduction of plasma cholesterol. In addition, the synthesis of LDL receptor is increased, which results in the increased uptake of LDL from the blood and a reduction of its level in plasma. One study performed on subjects with metabolic syndrome found that administration of colesevelam for 8 weeks resulted in a reduced fasting total plasma FFA concentration but an elevated postprandial total plasma FFA concentration [235].

Thiazolidinediones (e.g., darglitazone, rosiglitazone and pioglitazone) are hyperglycaemic agents that activate PPARγ [208,259]. These agents regulate the storage and uptake of FFAs and glucose in adipose tissue [259]. Numerous studies have investigated the effects of long-term administration (2 weeks to 3.5 years) of these drugs [144,236,237,238,239,240,241,242,243,244,245,246,247,248,249]. Most have found unchanged levels of total plasma FFAs in subjects with insulin resistance, glucose intolerance, T2DM or metabolic syndrome [144,241,242,243,248] or a reduction in total plasma FFA levels in healthy subjects or in subjects with insulin resistance or T2DM [236,237,238,239,240,242,244,245,246,247,249].

It is known that n-3 PUFAs can impact on plasma lipid and FFA concentrations through the mechanisms described in the previous sections, including the activation of PPARα and PPARγ and the reduction of inflammation. One study has shown that the administration of n-3 PUFAs for 10 weeks to subjects with T2DM resulted in reduced fasting total FFA concentrations in plasma [250].

ETC-1002 inhibits adenosine triphosphate citrate lyase, which is responsible for the cleavage of citrate to oxaloacetate and acetyl-CoA [198]. Acetyl-CoA is required for FFA and cholesterol synthesis. In a study carried out on subjects with hypercholesterolemia, it was found that administration of ETC-1002 for 8 weeks had no effect on total plasma FFA concentration [251].

The effects of different combinations of those drugs on total plasma FFA concentration have also been compared with the effect of the drugs on their own. Studies have examined the effects of metformin and rosiglitazone (a thiazolidinedione drug) on subjects with T2DM [252], fenofibrate and rosiglitazone on healthy subjects [253] and on subjects with T2DM [254], simvastatin and plant stanols on subjects with metabolic syndrome [255], atorvastatin and MBX-8025 on subjects with dyslipidaemia [256], simvastatin and ezetimibe on subjects with hypercholesterolemia [257] and rosuvastatin and ezetimibe on subjects with T2DM [258]. All these studies reported that the drug combinations reduced total plasma FFA concentrations more than each of the drugs were able to on their own, with the exception of the simvastatin and plant stanols combination which had no effect on total plasma FFA concentration.

### 6.2. Changes in Specific Plasma FFA Concentrations

In addition, it is interesting to consider how lipid-lowering drugs impact on specific FFAs or classes of FFAs. Table 4 summarises the results of studies examining how n-3 PUFAs and thiazolidinediones affect the plasma concentrations of specific FFAs. In addition, a study in which plasma FFAs were measured 4 h after an infusion of reconstituted HDL has been included. Table 5 details the changes in plasma FFA concentrations found in those studies. 

**Table 4:** List of studies having measured the concentrations of individual FFA species in the blood from subjects treated with lipid-lowering drugs and characteristics of the populations studied.

Drug type	Reference	Subjects	Drug Treatment	Types of Results
n-3 fatty acid	Conquer et al., 1998 [260]	22 healthy subjects	Placebo controlled study. Low docosahexaenoate 0.75 g/day or high docosahexaenoate 1.50 g/day for 42 days.	% total serum FFA
	Conquer et al., 1999 [261]	19 healthy men	Randomised, controlled, parallel group study. Seal-oil 1 g/day (1.3 g eicosapentaenoate, 1.7 g docosahexaenoate, and 0.8 g docosapentaenoate per day) for 42 days.	Serum FFA conc.
	Barre et al., 2016 [262]	32 T2DM subjects	Randomised, double-blind, controlled, parallel group. Flaxseed oil (60 mg of α-linolenate/ kg/day) for 3 months.	% total serum FFA
Thiazolidinediones	Yi et al., 2007 [98]	10 subjects with abdominal obesity and T2DM	Rosiglitazone (amount unknown), FFA measured 2, 7, 9 and 14 weeks after, compared to baseline.	Plasma FFA conc.
Reconstituted HDL infusions	Drew et al., 2011 [263]	13 T2DM patients	Randomised, double-blind, placebo-controlled, cross-over study. Reconstituted HDL infusion 80 mg/kg, FFA measured after 4 h, compared to a placebo given to same patients.	Plasma FFA conc.

**Table 5:** Changes in FFA concentrations measured in blood from subjects treated with lipid-lowering drugs. Elevated FFA concentrations are indicated with ↑, lower concentrations with ↓, unchanged concentrations with =. The numbers in brackets represent the number of weeks during treatment at which the measurement where made.

FFA Species	Altered Plasma FFA Conc. in T2DM after Lipid-Lowering Drug Treatment				
	Conquer et al. 1998 [260]	Conquer et al. 1999 [261]	Barre et al. 2016 [262]	Yi et al. 2007 [98]	Drew et al. 2011 [263]
Total FFAs				=	
C12:0, lauric acid			=	=	
C14:0, myristic acid	=		=	=	↑
C15:0, pentadecanoic acid				=	↑
C16:0, palmitic acid	=	=	=	=	↑
C18:0, stearic acid	=	=	=	↓ (14)	↑
C19:0, nonadecylic acid					↑
C20:0, arachidic acid				=	
C24:0, lignoceric acid				=	
C14:1n-5, myristoleic acid	=		=		
C16:1n-7, palmitoleic acid	=	=	=	=	
C16:1n-9*, cis*-7 hexadecenoic acid				=	
C18:1n-9, oleic acid (OA)	=	=	=	=	↑
C18:1n-7, *cis*-vaccenic acid				=	
C18:2n-6, linoleic acid (LA)	=	↓	=		
C18:3n-6, γ-linolenic acid (GLA)				↓ (14)	
C18:3n-3, α-linolenic acid (ALA)	=	=	↑	↓ (7)	↑
C20:3n-6, dihomo-γ-linolenic acid (DGLA)				=	=
C20:4n-6, arachidonic acid (ARA)	=	↓	=	=	=
C20:5n-3, eicosapentaenoic acid (EPA)	=	↑	↑	=	=
C22:4n-6, adrenic acid				↓ (2)	
C22:5 n3, docosapentaenoic acid (DPA)	=	↑		=	↑
C22:5n-6, osbondic acid	=				
C22:6n-3, docosahexaenoic acid (DHA)	↑	↑	=	↓ (9, 14)	ns
C14:1 *trans* -n-5*, trans*-myristoleic acid			=		
C16:1 *trans*-n-7, *trans*-palmitoleic acid			=		
C18:1 *trans-*n-7*,* vaccenic acid			=		
C18:1 *trans-*n-9, elaidic acid			=		
C18:2 *trans*-n-7 *cis*-n-9, rumenic acid					↑
C18:2 *trans*-n-6, linolelaidic acid			ns		

The effects of the administration of n-3 PUFAs on plasma FFA concentrations have been investigated in three studies. Two studies authored by Conquer et al. examined healthy subjects for 42 days [260,261], while Barre et al. studied a cohort of subjects with T2DM for 3 months [262]. They found that the n-3 PUFA-based drugs, unsurprisingly, resulted in an increase in the corresponding plasma n-3 FFAs constituting the drugs, including ALA, EPA and docosapentaenoic acid (DPA; C22:5) or DHA. The concentrations of other FFAs examined were unchanged, except for the n-6 PUFAs LA and ARA, which were found to be reduced in one of the Conquer et al. studies [261]. 

One study by Yi et al. has examined the effect of rosiglitazone on plasma FFA concentrations in subjects with abdominal obesity and T2DM up to 14 weeks after treatment [98]. The study revealed that the drug led to differing degrees of reduction in plasma FFA concentrations depending on the length of treatment. A reduction in the concentration of the n-6 adrenic acid was observed at week 2. At week 7 only ALA was reduced. DHA was reduced at week 9, and stearic acid, GLA and DHA at week 14.

Given that high LDL and low HDL plasma concentrations are risk factors for cardiovascular complications and LDL-reducing therapies are used in management of such conditions, the potential of using HDL-raising therapies has been evaluated [198]. Drew et al. have examined the effect of an infusion of reconstituted HDL (made of apolipoprotein AI isolated from pooled human plasma and phosphatidylcholine isolated from soy bean) on plasma FFA concentrations in a T2DM cohort [263]. The effect of the infusion on the total plasma FFA concentration is unknown but it led to an increase in many plasma FFAs in the short term (4 h after the infusion). These included the saturated FFAs, myristic acid, pentadecanoic acid, palmitic acid, stearic acid and nonadecylic acid (C19:0) and the unsaturated FFAs OA, ALA and DPA. Further studies are necessary to investigate the long-term effects of HDL infusion treatment on plasma FFA concentrations.

## 7. Conclusions and Future Perspectives

FFAs impact upon multiple physiological processes, and their effects depend on their chemical constitution. However, it has been pointed out that simple categorisations are often not appropriate to understand their physiological effects [264]. The picture becomes even more complicated when attempting to correlate fatty acid intake through diet with human health outcomes. Analysis of associations between dietary patterns and results from interventional studies suggest that besides ensuring a good balance between various fat types in the diet, endogenous de-novo synthesis of palmitate from excess carbohydrates should also be considered.

The evidence from in vitro cell culture and animal studies regarding harmful effects of elevated levels of palmitic acid and related SFAs is overwhelming, and our understanding of the pathways involved is quite advanced. Notably, an excess of plasma saturated FFAs causes dysregulation of satiety, an increase in inflammation and insulin resistance in most tissues, and a dysregulation of FFA storage by adipocytes that further increases plasma FFAs and dysregulates other plasma lipids. This results in the accumulation of harmful quantities and forms of fat in other tissues, leading to lipotoxicity, with downstream complications including β-cell apoptosis, non-alcoholic fatty liver disease and cardiovascular disorders. 

According to epidemiological studies, replacing SFAs with either MUFAs or PUFAs in the diet is principally beneficial, although it is often unclear whether the type of FA or other components of the respective source are more relevant in terms of health effects. A large body of work has been dedicated to long-chain n-3 PUFAs [69,71,128,134,265,266], whilst less information is available for MUFAs [145,267] and n-6 PUFAs [208]. In cell culture and animal studies, both OA and n-3 PUFAs have been shown to exert a protective effect against many of the SFA-mediated mechanisms and positively impact on inflammation and insulin sensitivity. However, whilst n-3 FFAs decrease plasma TAG in humans, and have hence beneficial effects due to the reduction of systemic inflammation, they have not been shown to confer the expected effects on insulin resistance [134,268]. In terms of diet, only plant-derived n-3 have shown a clear association with reduced T2DM risk [75]. Nonetheless, marine n-3 FFAs and related diets may offer beneficial effects to mitigate T2DM co-morbidities such as cardiovascular disease [269].

Although n-6 FFAs are often thought of as precursors to pro-inflammatory agents, this view is also too simplistic. The n-6 PUFAs LA and ARA are also precursors of anti-inflammatory agents, and indeed, there is no evidence from epidemiological studies that increased intake of LA or ARA promotes inflammation [79]. There is clearly a call for doubling efforts to understand their modes of action in development of T2DM. Similarly, association with T2DM risk, health effects, and cellular mechanisms are not sufficiently well studied in the case of MUFAs, despite more recent efforts to understand the benefits of the “Mediterranean diet” [35,49] which is rich in the n-9 MUFA OA.

One of the most exciting recent additions to the field under review concerns the interactions between diet, FFAs and the gut microbiome, with the latter on the one hand capable of producing healthy short-chain FAs, but also potentially contributing to chronic inflammation through the production of bacterial endotoxin. Manipulating the microbiome, e.g., through changes in diet [158], may offer drug-free ways to prevent or mitigate T2DM, with FFAs playing their part in this interplay. 

T2DM is often accompanied by dyslipidaemia, which includes elevated plasma FFAs which affect all major organs and tissues. Reversing these changes may be important for the management and treatment of T2DM and its co-morbidities. Many of the current lipid-lowering drugs, including n-3 FFAs supplements, have been shown to positively impact on total plasma FFAs, but further investigation is required as to the effects of those drugs in subjects with T2DM. This includes how they influence the plasma concentrations of specific FFAs in the long-term.

## Abbreviations

ALA (α-linolenic acid); ARA (arachidonic acid); AMPK (5’ AMP-activated protein kinase); CCK (cholecystokinin); DGLA (dihomo-γ-linolenic acid); DHA (docosahexaenoic acid); DTA (docosatetraenoic acid); EPA (eicosapentaenoic acid); ER (endoplasmic reticulum); FA (fatty acid); FABP (fatty-acid binding protein); FAT (fatty acid translocase); FFA (free fatty acid); GLA (γ-linolenic acid); GLUT-4 (glucose transporter type-4); (GPCR (G-protein coupled receptor); HDL (high density lipoprotein); HMG-CoA (3-hydroxy-3-methyl-glutaryl-coenzyme A); IKK (inhibitor of κB kinase); IKKβ (IκB kinase β); JNK (c-Jun N-terminal kinase); LA (linoleic acid); LDL (low density lipoprotein); LPS (lipopolysaccharides); MCFA (medium-chain fatty acid); MUFA (monounsaturated fatty acid); NLPR3 (NACHT, LRR and PYD domains-containing protein 3); OA (oleic acid); PPAR (peroxisome proliferator-activated receptors); PUFA (polyunsaturated fatty acid); RCT (randomly-controlled clinical trial); ROS (reactive oxygen species); SCFA (short-chain fatty acid); SFA (saturated fatty acid); T2DM (type-2 diabetes mellitus); TAB1 (growth factor β-activated kinase 1-binding protein); TAG (triacylglyceride), TAK1 (growth factor β-activated kinase 1); TLR (toll-like receptor); VLDL (very low-density lipoprotein).
